# Therapeutic vulnerability shaped by the microenvironment: multi-omics and AI biomarkers for precision surgical planning in gastrointestinal tumors

**DOI:** 10.3389/fcell.2026.1807136

**Published:** 2026-05-01

**Authors:** Da Guan

**Affiliations:** Department of General Surgery, First Medical Center of PLA General Hospital, Beijing, China

**Keywords:** artificial intelligence, gastric cancer, immunotherapy response, multi-omics integration, therapeutic vulnerability, tumor microenvironment

## Abstract

**Background:**

Therapeutic vulnerability in gastric cancer is profoundly influenced by the tumor microenvironment (TME), yet reliable and clinically actionable preoperative indicators remain insufficient.

**Methods:**

We developed and validated an artificial intelligence–driven multi-omics TME score (DLRS/TMEscore) by integrating CT-derived imaging features with transcriptomic, immunohistochemical, and molecular profiling. The score was evaluated for its associations with survival outcomes, benefit from adjuvant chemotherapy, and response to anti–PD-1 therapy.

**Results:**

The DLRS/TMEscore reproducibly stratified disease-free and overall survival across independent cohorts. Patients in the low-risk subgroup derived substantial benefit from adjuvant chemotherapy, whereas those in the high-risk subgroup demonstrated attenuated benefit. Among individuals receiving immunotherapy, the score enriched objective responders and predicted more durable clinical outcomes, outperforming established biomarkers including PD-L1 combined positive score (CPS) and microsatellite instability (MSI). In addition, DLRS/TMEscore correlated with multiple surgical parameters, such as operative complexity, resection margin status, nodal involvement, and postoperative recovery, indicating relevance in perioperative risk assessment.

**Conclusion:**

This AI-enabled multi-omics framework offers a robust and interpretable approach for characterizing microenvironment-defined therapeutic vulnerability, supporting preoperative risk stratification and individualized systemic treatment strategies in gastric cancer.

## 1 Introduction

As one of the most common malignant tumors worldwide, gastric cancer remains highly lethal and continues to be difficult to prevent and cure. In recent years, although standardized surgical procedures such as D2 lymph node dissection and the concept of total mesorectal excision have improved local control to some extent, patients still face substantial risks of postoperative recurrence and metastasis (Feng et al., 2024). A major clinical challenge is that even patients who undergo radical surgery and achieve histologically complete resection may subsequently develop peritoneal implantation or distant organ metastasis. These phenomena, to a certain degree, reflect limitations in current diagnostic and therapeutic strategies (Bao et al., 2024). In clinical practice, anatomical staging is widely adopted and primarily considers tumor size, the extent of invasion into surrounding tissues, regional lymph node involvement, and the presence or absence of distant metastasis. Although this macroscopic morphological classification is straightforward, it fails to account for the intrinsic biological heterogeneity among tumors (Jiang et al., 2023; Shi et al., 2024). Because molecular-level therapeutic vulnerabilities differ across individuals, traditional staging approaches cannot accurately predict treatment responses within a complex microenvironment, thereby limiting further advances in precision surgical planning for gastric cancer (George et al., 2024).

Tumor progression and drug resistance are largely driven by dynamic alterations within the microenvironment, which facilitate tumor evolution and the emergence of therapy-resistant niches. Gastric cancer should therefore be regarded not merely as a collection of malignant epithelial cells, but as an ecological system composed of cancer-associated fibroblasts, infiltrating immune cells, and a remodeled extracellular matrix. Recent research indicates that both the physical structure and biochemical composition of the tumor microenvironment provide natural protection for tumor cells, enabling them to exhibit remarkable adaptability and resistance under conventional chemotherapy or radiotherapy (Ji et al., 2024). Moreover, heterogeneity mediated by the microenvironment varies significantly across spatial regions. When matrix components are excessively accumulated or immune infiltration is suppressed, tumor sensitivity to treatment is further reduced. This variability highlights the importance of understanding therapeutic response through ecosystem-level interactions rather than focusing solely on tumor cells themselves (Li et al., 2024).

The rapid development of multi-omics technologies has provided strong support for integrative decoding of the tumor microenvironment at molecular, cellular, and tissue levels. The combined application of genomics, transcriptomics, and proteomics enables researchers to explore fundamental drivers of microenvironmental remodeling with unprecedented depth. However, multi-omics data are inherently high-dimensional and complex, making it challenging to extract biomarkers that can be readily translated into clinical practice (Xiang et al., 2024). Artificial intelligence, particularly deep learning, has emerged as an innovative solution to these challenges. By constructing advanced computational frameworks, AI can detect subtle feature variations within omics datasets and link them to clinically meaningful outcomes. This approach facilitates the identification of potential therapeutic targets and enhances the interpretation of complex treatment responses. The integration of AI and multi-omics is therefore transforming cancer diagnosis and treatment from experience-based practice toward precision computational medicine.

The present study focuses on the development and validation of a model termed the Deep Learning Multi-Omics Score (Sun et al., 2024). By integrating large-scale, multi-center clinical multi-omics data, the aim is to quantify therapeutic vulnerability in gastric cancer at the level of microenvironmental remodeling. This score is intended not only to serve as an independent prognostic indicator for disease-free survival (DFS) and overall survival (OS), but more importantly to function as a direct decision-support tool for surgical planning. Based on this score, clinicians may achieve improved preoperative biological stratification of patients. For individuals with higher vulnerability scores, it may be necessary to expand the surgical resection margin or incorporate targeted neoadjuvant strategies, thereby enabling more precise and individualized perioperative management (Bai et al., 2024).

## 2 Methods

### 2.1 Patient stratification and multi-center cohort design

**FIGURE 1:**
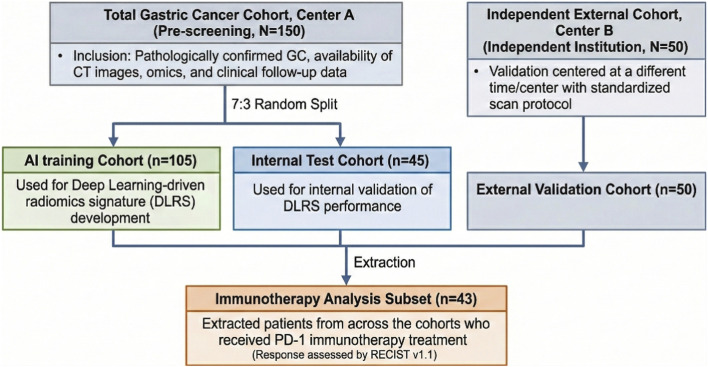
Cohort flowchart and study design for patient allocation.

This study utilized a multi-center retrospective cohort design (Figure 1) to develop and validate the AI-driven multi-omics model. The primary cohort consisted of 150 consecutive gastric cancer patients who received standardized treatment at the Tumor Center of First Medical Center of PLA General Hospital and satisfied the criteria for combined imaging-multi-omics analysis. To facilitate model development and internal evaluation, this primary cohort was randomly partitioned into a training cohort (n = 105) and an internal validation cohort (n = 45) at a 7:3 ratio. To rigorously assess model generalizability, an independent external validation cohort comprising 50 patients was retrospectively collected from Peking University Cancer Hospital, National Cancer Center, Beijing, China, using identical imaging acquisition protocols and harmonized clinical inclusion criteria to ensure cross-institutional consistency. Strictly adhering to the same imaging and clinical inclusion criteria. Furthermore, to evaluate the predictive value of the TMEscore in the context of immune checkpoint blockade, an immunotherapy subset of 43 patients (derived from the aforementioned cohorts who explicitly received anti-PD-1 therapy) was established for subsequent response analyses. The study complied with institutional ethics committee requirements, and all data were de-identified prior to scientific analysis. To ensure consistency and comparability for model training and clinical outcome evaluation, unified clinical pathways and data integrity standards were predefined during the case screening period (Massa et al., 2024). Baseline clinical characteristics, tumor biological features, and treatment strategies were categorized at this stage to provide a structured foundation for subsequent interpretation of microenvironment-driven therapeutic vulnerability.

The inclusion criteria were as follows: ① pathological confirmation of gastric adenocarcinoma; ② completion of preoperative evaluation with an eligible contrast-enhanced CT examination, including the portal venous phase and image quality sufficient for subsequent segmentation and feature extraction; ③ availability of pathological staging information (pT, pN, and TNM) together with detailed invasive characteristics, such as lymphovascular invasion (LVI), perineural invasion (PNI), and resection margin status; and ④ accessible postoperative follow-up data enabling calculation of disease-free survival (DFS) and overall survival (OS). The exclusion criteria were: ① significant imaging artifacts or severe inconsistency in slice thickness or contrast timing that would compromise reliable feature extraction; ② prior gastric surgery or coexisting malignancies interfering with interpretation of the primary lesion; and ③ absence of essential clinical indicators, including staging information, treatment exposure, or primary outcomes, which would preclude stratified or adjusted analyses. This screening process aimed to enhance internal consistency of the cohort and minimize systematic bias in AI modeling and survival inference arising from heterogeneity in imaging and clinical data.

Regarding patient stratification, the study emphasized intervenable therapeutic vulnerability, and variables influencing preoperative decision-making and perioperative strategy selection were defined as primary strata. First, a framework for tumor burden and recurrence risk was constructed according to pathological stage and local invasion status (pT, pN, and TNM). Second, biological heterogeneity was characterized based on histological subtype and differentiation, including Lauren classification, differentiation grade, and the presence of signet-ring cell components. Third, exposure stratification was performed according to treatment pathways, specifically whether patients received neoadjuvant or adjuvant chemotherapy (Formica et al., 2024). For those undergoing immunotherapy, exposure status and evaluation time points were further specified to investigate the predictive and interactive effects of the AI-driven microenvironment score across therapeutic strategies. Fourth, key clinical indicators and routine biomarkers, including CEA, CA19-9, HER2, PD-L1 CPS, and MSI/EBV, were incorporated as covariates to establish a risk-adjusted and subgroup interpretation framework aligned with real-world clinical application (Gou et al., 2024).

Table 1 systematically summarizes baseline demographic features, tumor location and size, pathological stage, invasion-related variables, surgical approaches, and perioperative treatment information for the study population.

**TABLE 1:** Patient demographics and tumor characteristics for gastric cancer cohorts.

Characteristic	Overall (n = 150)
Age, years	62.1 ± 11.2
Sex	
Male	98 (65.3)
Female	52 (34.7)
Body mass index (BMI), kg/m^2^	23.6 ± 3.4
<18.5	12 (8.0)
18.5–24.9	98 (65.3)
≥25.0	40 (26.7)
ECOG performance status	
0	104 (69.3)
1	43 (28.7)
2	3 (2.0)
Comorbidities	
Hypertension	44 (29.3)
Diabetes mellitus	23 (15.3)
Coronary artery disease	17 (11.3)
Lifestyle factors	
Smoking status	
Never	83 (55.3)
Former	29 (19.3)
Current	38 (25.3)
Alcohol consumption (current)	41 (27.3)
*Helicobacter pylori* status	
Positive	73 (48.7)
Negative	57 (38.0)
Unknown/not tested	20 (13.3)
Primary tumor location	
Upper third	32 (21.3)
Middle third	53 (35.3)
Lower third	47 (31.3)
Diffuse/overlapping	18 (12.0)
Tumor size (largest diameter), cm	4.4 (3.0–6.1)
Histology (Lauren classification)	
Intestinal	66 (44.0)
Diffuse	62 (41.3)
Mixed	22 (14.7)
Histologic differentiation	
Well differentiated	12 (8.0)
Moderately differentiated	58 (38.7)
Poorly differentiated/signet-ring	80 (53.3)
Pathological T stage (pT)	
pT1	22 (14.7)
pT2	28 (18.7)
pT3	56 (37.3)
pT4	44 (29.3)
Pathological N stage (pN)	
pN0	44 (29.3)
pN1	34 (22.7)
pN2	36 (24.0)
pN3	36 (24.0)
AJCC TNM stage (8th edition)	
I	28 (18.7)
II	46 (30.7)
III	60 (40.0)
IV	16 (10.7)
Lymphovascular invasion (LVI)	49 (32.7)
Perineural invasion (PNI)	37 (24.7)
Resection margin status	
R0	138 (92.0)
R1/R2	12 (8.0)
Surgical approach	
Open	86 (57.3)
Laparoscopic/robotic	64 (42.7)
Extent of gastrectomy	
Distal gastrectomy	68 (45.3)
Total gastrectomy	58 (38.7)
Proximal gastrectomy	12 (8.0)
Palliative/bypass procedures	12 (8.0)
D2 lymphadenectomy performed	131 (87.3)
Neoadjuvant chemotherapy	36 (24.0)
Adjuvant chemotherapy (planned/received)	89 (59.3)
Baseline serum CEA, ng/mL	3.8 (1.9–7.2)
CEA >5.0 ng/mL	41 (27.3)
Baseline serum CA19–9, U/mL	19.6 (8.4–42.7)
CA19-9 > 37 U/mL	33 (22.0)
Biomarkers	
HER2 status	
Negative	119 (79.3)
Equivocal	9 (6.0)
Positive	22 (14.7)
PD-L1 CPS category	
<1	64 (42.7)
1–9	58 (38.7)
≥10	28 (18.7)
MSI status	
MSI-H	18 (12.0)
MSS/MSI-L	132 (88.0)
EBV-positive tumor	10 (6.7)

### 2.2 Multi-omics data integration and TME profiling

**FIGURE 2:**
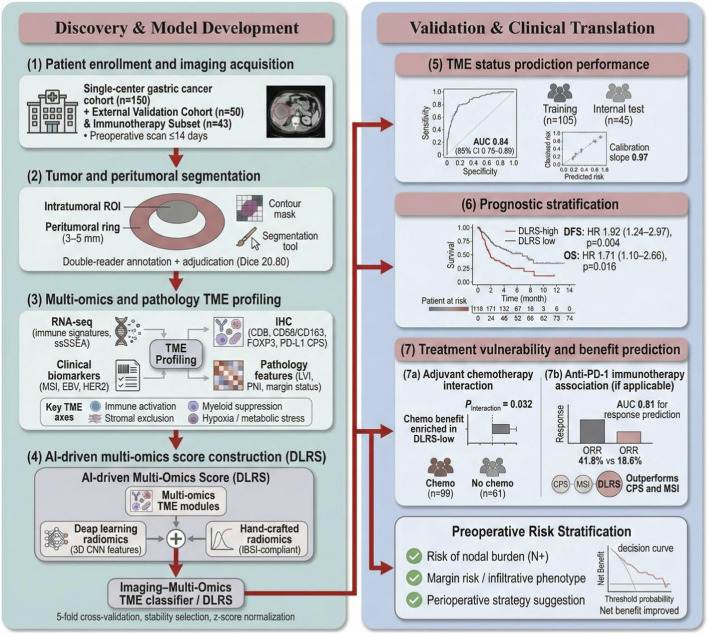
Study design for the development and validation of an AI-driven multi-omics model for tumor microenvironment assessment and treatment outcome prediction in gastric cancer.

A multi-omics spectrum of the tumor microenvironment (TME) was constructed in association with imaging-derived deep learning phenotypes, incorporating key dimensions such as immune cell infiltration, stromal barriers and remodeling, myeloid-mediated immune suppression, and hypoxic metabolic adaptation in gastric cancer. The workflow encompassed patient enrollment, preoperative contrast-enhanced CT acquisition, tissue-based molecular analyses, and cross-modality integration, thereby forming a closed-loop framework of mutual verification between imaging phenotypes and molecular microenvironmental evidence. An overview of the study design and major analytical steps is presented in Figure 2. The majority of multi-omics data were derived from tumor tissue transcriptomics, quantitative immunohistochemistry, and routinely reported clinical molecular markers. These data were harmonized and integrated at the patient level to support subsequent construction of the deep learning–based risk score (DLRS) and interpretation of therapeutic vulnerabilities (Huang et al., 2025).

The primary source of tissue material consisted of surgical resection specimens. When preoperative biopsy samples were required, they were incorporated into the analysis, and sensitivity analyses were conducted to ensure data consistency. Transcriptomic datasets underwent standardized quality control and expression normalization procedures. Low-abundance genes were removed prior to regularization, and batch effects were assessed to minimize systematic bias. Immunohistochemistry employed a standardized antibody panel to quantitatively evaluate the distribution of cellular components within the TME, including immune checkpoint–related markers such as CD8^+^ T-cell infiltration, macrophage-associated markers, regulatory T-cell indicators, and PD-L1 expression. Clinical molecular variables, including MSI, EBV, and HER2 status, were incorporated as anchor features reflecting immune–inflammatory phenotypes and potential therapeutic sensitivity (Wang et al., 2025). Through standardized preprocessing and unified coding of multi-source data, a patient-level TME feature matrix was generated, providing traceable molecular evidence to support biological interpretation following output from the imaging model.

The construction of the tumor microenvironment (TME) signature employed a modular integration strategy to project transcriptome-inferred immune infiltration and pathway activity, immunohistochemical quantitative indices, and clinical molecular markers into a unified representational space. Specifically, the immune activation dimension emphasized T-cell inflammation–related signaling and antigen-presenting capacity. The stromal and immune exclusion dimensions focused on TGF-β–associated pathways, epithelial–mesenchymal transition (EMT), and extracellular matrix remodeling. The myeloid suppression dimension captured myeloid programs associated with immunoregulatory inhibition, whereas the hypoxia and metabolic dimension characterized features related to oxygen deprivation and metabolic reprogramming (Tashireva et al., 2025). Cross-omics concordance was subsequently evaluated through directional consistency testing between transcriptome-inferred immune infiltration and immunohistochemical measurements to ensure mutual validation across modalities. The overall TMEM profile and its key indicators are systematically summarized in Table 2.

**TABLE 2:** Tumor microenvironment profile based on multi-omics analysis in gastric cancer.

TME domain/feature	Assay (omics layer)	Metric/definition	Overall (n = 150)
Immune infiltration (inferred)	RNA-seq (deconvolution)	CD8^+^ T-cell fraction	0.087 (0.051–0.132)
	RNA-seq (deconvolution)	CD4^+^ T-cell fraction	0.064 (0.039–0.091)
	RNA-seq (deconvolution)	Treg fraction	0.029 (0.018–0.043)
	RNA-seq (deconvolution)	Macrophage (M2-like) fraction	0.112 (0.074–0.166)
	RNA-seq (deconvolution)	NK cell fraction	0.021 (0.011–0.036)
Immune activation signatures	RNA-seq (ssGSEA)	IFN-γ response score (z)	−0.06 ± 0.98
	RNA-seq (ssGSEA)	Cytolytic activity score (z)	0.08 ± 1.03
	RNA-seq (module score)	Antigen presentation (MHC-II) score (z)	−0.03 ± 0.96
	RNA-seq (module score)	T cell–inflamed signature score (z)	0.04 ± 0.99
Myeloid suppression/inflammation	RNA-seq (module score)	SPP1+ macrophage program (z)	0.11 ± 1.01
	RNA-seq (module score)	IL-6/JAK/STAT3 activity (z)	0.02 ± 0.94
	RNA-seq (module score)	Neutrophil recruitment program (z)	−0.07 ± 1.00
Stromal exclusion and remodeling	RNA-seq (ssGSEA)	TGF-β signaling score (z)	0.09 ± 0.97
	RNA-seq (ssGSEA)	EMT program score (z)	0.13 ± 1.04
	RNA-seq (module score)	CAF activation score (z)	0.06 ± 0.95
	RNA-seq (module score)	ECM remodeling score (z)	0.10 ± 1.02
Hypoxia and metabolism	RNA-seq (ssGSEA)	Hypoxia score (z)	0.05 ± 0.99
	RNA-seq (ssGSEA)	Glycolysis score (z)	0.12 ± 0.98
	RNA-seq (ssGSEA)	Oxidative phosphorylation score (z)	−0.08 ± 0.96
Pathology-based immune readouts	IHC (digital/semi-quant)	CD8^+^ density at invasive margin (cells/mm^2^)	168 (92–286)
	IHC (digital/semi-quant)	CD68^+^ density (cells/mm^2^)	214 (136–328)
	IHC (digital/semi-quant)	CD163+/CD68+ ratio (%)	41.6 ± 14.7
	IHC (semi-quant)	FOXP3+ density (cells/mm^2^)	52 (28–88)
Immune checkpoint marker	IHC	PD-L1 CPS, median (IQR)	3 (1–9)
	IHC	PD-L1 CPS category	
	​	<1	64 (42.7)
	​	1–9	58 (38.7)
	​	≥10	28 (18.7)
Molecular subtype markers	Clinical report	MSI-H	18 (12.0)
	Clinical report	EBV-positive	10 (6.7)
	Clinical report	HER2 positive	22 (14.7)
Integrated TME phenotype (derived)	Multi-omics integration	Immune-active	46 (30.7)
	Multi-omics integration	Stromal-excluded	58 (38.7)
	Multi-omics integration	Myeloid-suppressed	34 (22.7)
	Multi-omics integration	Immune-desert	12 (8.0)

### 2.3 Develop-ment of the AI-driven multi-omics score

This study aimed to establish an AI-driven multi-omics scoring system termed the Deep Learning–Related Score (DLRS) to characterize the tumor microenvironment (TME) state of gastric cancer and to reveal its associated therapeutic vulnerability. The DLRS framework followed principles of dual translation and interpretability, seeking not only to achieve predictive performance for clinical outcomes and treatment responses, but also to evaluate its associations with key biological processes, including immune activation, stromal exclusion, and myeloid-mediated immunosuppression.

#### 2.3.1 CT preprocessing and deep learning feature extraction

To ensure analytical reproducibility, a standardized pipeline was implemented for CT imaging. Preoperative contrast-enhanced CT images (portal venous phase) were resampled to an isotropic resolution of 1.0 × 1.0 × 1.0 mm^3^. Image intensities were normalized using Z-score transformations, with the display window set to a width of 400 HU and a level of 40 HU. Intratumoral and peritumoral regions of interest (ROIs) were annotated using 3D Slicer software by two radiologists with over 5 years of experience, blinded to clinical outcomes. Discrepancies were adjudicated by a senior radiologist with 10 years of experience, and the inter-reader Dice similarity coefficient was ≥0.82.

For deep learning feature extraction, we employed a 3D ResNet-18 architecture as the backbone network. The model was trained using the AdamW optimizer with a learning rate of 1 × 10^−4^, a weight decay of 1 × 10^−5,^ and a batch size of 16. The Binary Cross Entropy (BCE) loss function was utilized for optimization. The training process was conducted over a maximum of 100 epochs, incorporating an early stopping strategy with a patience of 15 epochs to mitigate overfitting. The random seed was fixed at 42 to ensure computational reproducibility. Extracted 3D CNN features were subsequently integrated with transcriptomic and IHC modules to construct the final DLRS/TMEscore.

#### 2.3.2 Multi-omics feature integration and model construction

To address challenges related to high dimensionality and potential overfitting in omics data, a staged strategy was adopted, consisting of feature pre-screening, cross-validated stability selection, and interpretable model construction. After alignment of multi-omics features at the patient level, a unified feature matrix was generated for DLRS training.

During feature engineering, multi-source datasets, including transcriptomic and immunohistochemical variables, were standardized. Continuous molecular scores and pathway signatures were normalized using z-scores to improve comparability across dimensions. Categorical variables were transformed into dummy variables. Given the presence of missing values in certain data elements, clinically informed imputation strategies were applied to complete the dataset, and the impact of imputation on model robustness was further examined through sensitivity analyses. Subsequently, a two-step pre-screening procedure was performed at both biological and statistical levels. Features closely associated with tumor immune inflammation, stromal remodeling, hypoxic metabolism, and myeloid suppression were retained, while highly collinear or redundant variables were excluded to enhance model generalizability and interpretability.

Regarding model construction, DLRS adopted a supervised learning framework based on DFS (Depth First Search) results, with disease-free survival time defined as the optimization target. Hierarchical cross-validation was introduced during model training to stabilize feature selection. Specifically, feature importance was estimated through repeated resampling procedures. During iterative cross-validation for determining optimal model structures and parameters, variables demonstrating consistent generalization performance were retained. These selected features were subsequently integrated into the foundational architecture of the DLRS. To enhance clinical interpretability, core variables were categorized into three functional axes: immune activation, stromal exclusion, and myeloid suppression, which were then aggregated into a continuous output score. Higher scores indicated a microenvironment characterized by stronger immune exclusion or immunosuppression, typically associated with insufficient therapeutic response and increased recurrence risk, thereby reflecting greater therapeutic vulnerability. Conversely, lower scores suggested a microenvironment more permissive to immune activation and favorable antitumor responses.

To facilitate clinical applicability and verify translational value, DLRS was further evaluated in combination with conventional clinicopathological biomarkers. The score was incorporated into survival models as an independent predictor and simultaneously co-modeled with treatment exposure variables to assess its potential role in stratifying benefit from adjuvant chemotherapy or immunotherapy through interaction analyses. Finally, the AI-driven multi-omics biomarkers, together with their associations with outcomes and therapeutic responses, are summarized in Table 3, providing multi-level visualization of the origins, operational definitions, and implications of DLRS components in defining therapeutic vulnerability.

**TABLE 3:** AI-driven biomarker identification from multi-omics data for predicting treatment vulnerability in gastric cancer.

Biomarker (AI-selected feature)	Omics layer/assay	Operational definition	Vulnerability implication (high level)	Adjusted association with DFS (HR, 95% CI)	Treatment interaction signal
T cell–inflamed signature score	Transcriptomics (RNA-seq)	Weighted score of cytotoxic/IFN-γ–related genes	Lower vulnerability (immune-active, better control)	0.71 (0.56–0.90)	Anti–PD-1 benefit enriched (trend; OR* 1.84, 1.05–3.24)
IFNG pathway activity	Transcriptomics	ssGSEA IFN-γ response pathway	Lower vulnerability	0.76 (0.60–0.96)	Anti–PD-1 benefit enriched (OR* 1.67, 1.01–2.93)
Antigen presentation score (MHC-II)	Transcriptomics	HLA-DRA/HLA-DRB1/CD74 module score	Lower vulnerability	0.79 (0.63–0.99)	Anti–PD-1 benefit enriched (OR* 1.55, 0.98–2.69)
CD8^+^ T-cell density	Pathology (IHC)	CD8^+^ cells per mm^2^ at invasive margin	Lower vulnerability	0.74 (0.58–0.95)	Anti–PD-1 benefit enriched (OR* 1.61, 0.99–2.88)
PD-L1 CPS (≥10 vs. <10)	Pathology (IHC, CPS)	Standard CPS category	Lower vulnerability (immune-reactive context)	0.86 (0.66–1.12)	Anti–PD-1 response enriched (OR* 1.92, 1.08–3.61)
MSI-H (yes vs. no)	Genomics/pathology	MSI testing (clinical report)	Lower vulnerability (immune-sensitive subset)	0.69 (0.44–1.07)	Anti–PD-1 response enriched (OR* 2.21, 1.11–4.56)
TGF-β/EMT program score	Transcriptomics	TGF-β signaling + EMT composite score	Higher vulnerability (immune exclusion, invasion)	1.42 (1.12–1.79)	Reduced chemo benefit (P_interaction† 0.032)
ECM remodeling score	Transcriptomics	COL1A1/COL3A1/MMP2/POSTN module	Higher vulnerability	1.36 (1.08–1.71)	Reduced chemo benefit (P_interaction† 0.041)
CAF activation score	Transcriptomics	FAP/PDGFRB/ACTA2 module	Higher vulnerability	1.33 (1.05–1.69)	Reduced chemo benefit (P_interaction† 0.048)
SPP1+ macrophage program	Transcriptomics	SPP1/APOE/LGALS3 module score	Higher vulnerability (immunosuppression)	1.39 (1.10–1.77)	Anti–PD-1 benefit attenuated (P_interaction‡ 0.027)
M2-like macrophage ratio	Pathology (IHC)	CD163+/CD68+ proportion (%)	Higher vulnerability	1.31 (1.02–1.68)	Anti–PD-1 benefit attenuated (P_interaction‡ 0.044)
Hypoxia score	Transcriptomics	CA9/VEGFA/SLC2A1 module	Higher vulnerability (aggressive biology)	1.28 (1.01–1.62)	Reduced chemo benefit (P_interaction† 0.039)

### 2.4 Statistical modeling

A prespecified statistical analysis framework was applied for model construction and inference. Continuous variables were summarized as mean ± standard deviation or median (interquartile range), depending on their distribution. Categorical variables were presented as counts and proportions. Patients were classified into high- and low-risk groups according to the predefined DLRS threshold. Between-group comparisons were conducted using parametric or nonparametric tests as appropriate, while categorical variables were analyzed using the chi-square test or Fisher’s exact test. All statistical tests were two-sided, with a significance level set at 0.05. Throughout the analytical process, consistent definitions of variables and outcome measurements were maintained to minimize potential selection bias.

The primary endpoints were disease-free survival (DFS) and overall survival (OS). Kaplan-Meier methods were used to generate survival curves and to compare differences between groups. Associations between DLRS and DFS or other outcomes of interest were evaluated using Cox proportional hazards models. The modeling strategy progressed from univariable analysis to multivariable adjustment, incorporating clinically relevant covariates; under these conditions, the independent contribution of DLRS could no longer be interpreted in isolation. The proportional hazards assumption was tested, and appropriate corrective strategies were implemented when violations were detected. To examine the stratification capacity of DLRS across different therapeutic pathways, interaction terms between DLRS and treatment modalities were introduced. When treatment allocation bias required adjustment, propensity score matching or weighting approaches were applied, followed by assessment of post-adjustment covariate balance. The robustness of analytical findings was confirmed through repeated verification procedures.

Model performance was evaluated from multiple perspectives, including discrimination, calibration, and clinical utility. For survival outcomes, concordance indices (C-indices) and bootstrap-derived confidence intervals were calculated, whereas area under the curve (AUC) metrics were reported for categorical endpoints. Calibration performance was assessed using calibration plots and error curves. Clinical value was further quantified through decision curve analysis to estimate net benefit. Model stability was examined by evaluating consistency after covariate adjustment, re-estimation within stratified subgroups, and alternative handling of missing data. To determine generalizability, internal validation was performed using cross-validation or bootstrap resampling methods (Chen et al., 2025).

## 3 Results

### 3.1 Performance and robustness of the AI-driven model

**FIGURE 3:**
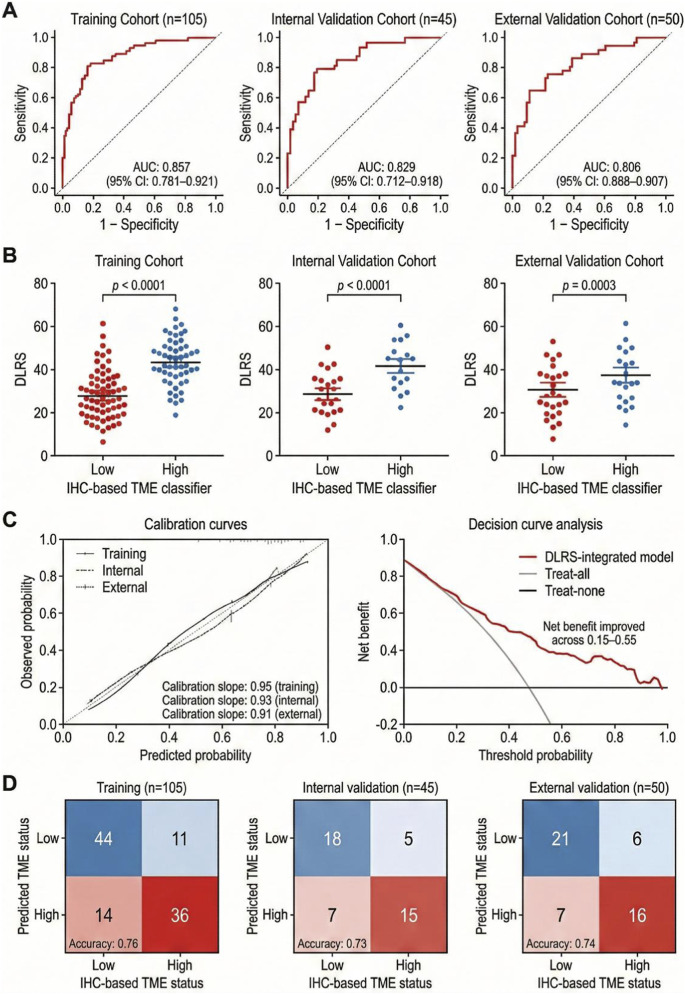
Performance of the AI-driven multi-omics model in tumour microenvironment evaluation across training, internal validation and external validation cohorts for gastric cancer. **(A)** Receiver operating characteristic (ROC) curves showing DLRS-based TME classification performance in the training, internal validation, and external validation cohorts. **(B)** Distribution of DLRS values stratified by the IHC-based TME classifier (Low vs. High) across cohorts. **(C)** Calibration curves assessing model calibration and decision curve analysis assessing clinical utility. **(D)** Confusion matrices summarizing the agreement between DLRS-predicted TME status and IHC-defined TME status in each cohort.

This study first evaluated the discriminative performance of the AI-driven multi-omics model for classifying tumor microenvironment (TME) states in gastric cancer. Consistency assessments were conducted across the training, internal validation, and external validation cohorts. At the whole-slide level, immune expression differences remained observable across data partitions. Overall, clear distinctions between high- and low-TME groups were identified based on immunohistochemical staining. The central performance metrics and multidimensional robustness are summarized in Figure 3.

Discriminative ability is illustrated in Figure 3A through receiver operating characteristic (ROC) curves for the three cohorts. The training cohort curve is located closer to the upper-left region of the plot, indicating strong classification performance. Although the area under the curve (AUC) values in the internal and external validation cohorts were lower than those in the training cohort, the overall curve patterns and separation capacity remained relatively stable. Continuous identification of TME states by DLRS across different cohorts provided the basis for subsequent prognostic stratification and treatment benefit evaluation.

Hierarchical consistency between DLRS and biologically defined TME categories is presented in Figure 3B. Across all cohorts, the mean DLRS values in the high-TME group were significantly greater than those in the low-TME group, with statistically significant differences observed. Notably, the external validation cohort demonstrated the same separation direction and comparable distribution intervals as the training cohort, despite the smaller sample size. These findings indicate stable correspondence between DLRS and histologically characterized microenvironmental states.

Model calibration and potential clinical applicability are presented in Figure 3C. The calibration curves generally approximated the reference diagonal, indicating agreement between predicted probabilities and observed outcomes. This supports the feasibility of applying DLRS as a continuous variable for individualized risk assessment and stratified decision-making. Decision curve analysis further demonstrated that, within clinically relevant threshold ranges, the net benefit of the DLRS-based strategy exceeded those of uniform intervention or non-intervention approaches. Together, these results describe the statistical performance and decision-support characteristics of the model.

Classification reliability is depicted in Figure 3D using a confusion matrix to compare DLRS-predicted TME categories with immunohistochemical subtypes. Overall, the model achieved satisfactory separation between the principal TME classes. Misclassification was primarily observed near category boundaries, where samples transitioned between high and low TME states. This pattern is consistent with the continuous nature of microenvironmental variation. These findings provide a quantitative reference for interpreting DLRS-based classification outputs.

### 3.2 Prognostic value of DLRS in long-term survival

**FIGURE 4:**
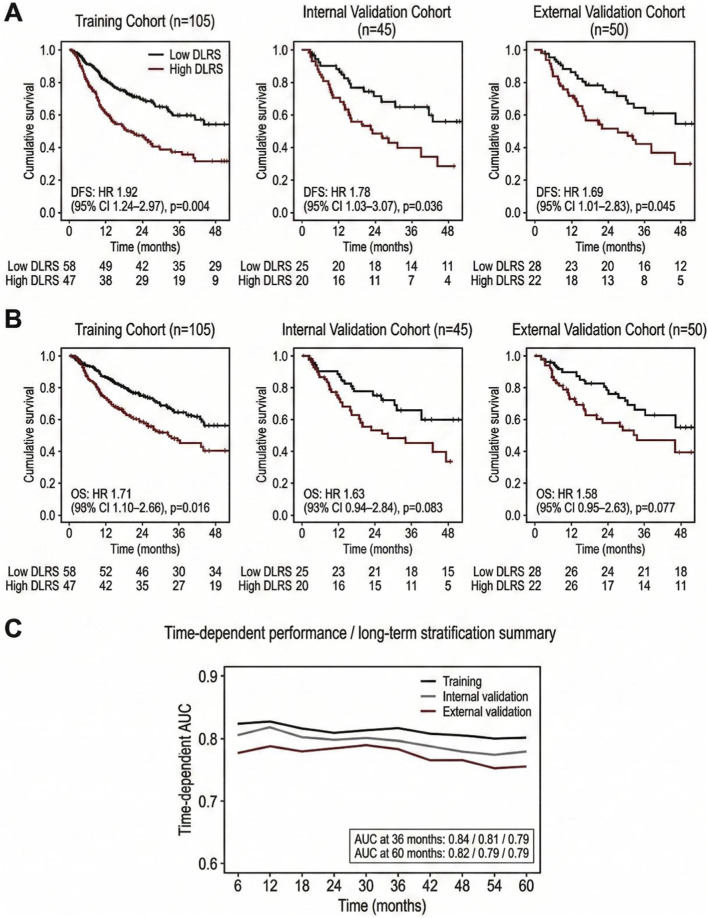
Kaplan-Meier analysis of disease-free survival (DFS), overall survival (OS), and time-dependent discrimination performance of the AI-driven multi-omics tumor microenvironment score (DLRS) in patients with gastric cancer. **(A)** Kaplan–Meier curves of DFS comparing high- and low-risk groups defined by DLRS in the training, internal validation, and external validation cohorts. The cutoff value separating the high- and low-risk groups was predefined based on the optimal Youden index derived exclusively from the training cohort and consistently applied to the validation sets. **(B)** Kaplan–Meier curves of OS for DLRS-defined high- and low-risk groups across the same cohorts. For both DFS and OS analyses, the median follow-up time was 41 months. Patients who were alive without recurrence or lost to follow-up were right-censored at the date of last clinical contact. **(C)** Time-dependent area under the curve (AUC) demonstrating the discrimination performance of DLRS at different follow-up times in the training, internal validation, and external validation cohorts.

A comprehensive evaluation was conducted to determine the stratification capability of the AI-assisted multi-omics tumor microenvironment score, DLRS, in predicting long-term outcomes in patients with gastric cancer, while also examining its association with clinical endpoints. Overall, DLRS demonstrated stable separation of recurrence and survival risks with consistent directional trends across cohorts, indicating its capacity to capture prognostic information related to the tumor microenvironment. As illustrated in Figure 4A, Kaplan–Meier curves for disease-free survival (DFS) showed that, in the training cohort, patients in the high-DLRS group experienced earlier and steeper declines, corresponding to increased recurrence risk. A similar separation pattern was observed in both internal and external validation cohorts. Although the magnitude of separation was reduced compared with the training cohort, the direction of stratification remained consistent and retained statistical or marginal significance. These findings demonstrate that DLRS maintains stratification ability across datasets and identifies a phenotype characterized by elevated recurrence susceptibility. Overall survival results are presented in Figure 4B, where DLRS also achieved risk separation. Compared with DFS, differences between OS curves were less pronounced. Nevertheless, across all cohorts, higher DLRS values were consistently associated with less favorable survival patterns. Temporal discrimination performance is further shown in Figure 4C. At successive follow-up intervals, DLRS maintained relatively stable discriminative capacity, with expected variation between training and validation sets but without evidence of performance deterioration. Together with the results observed for DFS and OS, DLRS demonstrated the ability to provide consistent prognostic stratification across both individual time points and extended follow-up durations.

Table 4 quantifies the associations between DLRS and multiple clinical endpoints. When modeled either as a continuous variable or as a categorical stratification factor, DLRS was associated with increased risks of disease-free survival (DFS) and overall survival (OS), and showed a positive relationship with early recurrence events. These results demonstrate the predictive capacity of DLRS across recurrence-related adverse outcomes.

**TABLE 4:** Correlation between AI-driven tumor microenvironment scores and clinical outcomes in gastric cancer.

Outcome/endpoint	Analysis set	Effect of DLRS (per 1-SD increase)	Effect of DLRS (high vs. Low)*	P value
Disease-free survival (DFS)	Overall cohort	HR 1.44 (1.18–1.76)	HR 1.92 (1.24–2.97)	0.004
Overall survival (OS)	Overall cohort	HR 1.31 (1.05–1.64)	HR 1.71 (1.10–2.66)	0.016
Recurrence within 24 months	Overall cohort	OR 1.38 (1.10–1.75)	OR 2.06 (1.05–4.10)	0.035
Death within 36 months	Overall cohort	OR 1.29 (1.01–1.66)	OR 1.81 (0.93–3.56)	0.081
Pathologic nodal metastasis (pN+)	Curative-intent surgery (n = 138)	OR 1.41 (1.12–1.79)	OR 2.18 (1.16–4.14)	0.015
Lymphovascular invasion (LVI)	Curative-intent surgery (n = 138)	OR 1.33 (1.06–1.68)	OR 1.89 (1.02–3.55)	0.043
Perineural invasion (PNI)	Curative-intent surgery (n = 138)	OR 1.27 (1.01–1.60)	OR 1.62 (0.88–3.03)	0.118
R1/R2 resection margin	Curative-intent surgery (n = 138)	OR 1.36 (0.98–1.91)	OR 2.04 (0.83–5.25)	0.122
Major postoperative complication (Clavien–Dindo ≥ III)	Curative-intent surgery (n = 138)	OR 1.18 (0.90–1.55)	OR 1.37 (0.71–2.64)	0.341
Objective response to anti–PD-1 (ORR)†	Immunotherapy subset (n = 43)	OR 1.61 (1.03–2.55)	OR 2.52 (1.02–6.44)	0.046
Progression-free survival under anti–PD-1 (PFS)†	Immunotherapy subset (n = 43)	HR 1.52 (1.08–2.15)	HR 1.88 (1.05–3.37)	0.033
DFS benefit from adjuvant chemotherapy (interaction)‡	Stage II–III, R0 (n = 94)	P_interaction = 0.032	Chemo benefit enriched in DLRS-low	0.032

### 3.3 Guiding adjuvant chemotherapy and surgical strategies

**FIGURE 5:**
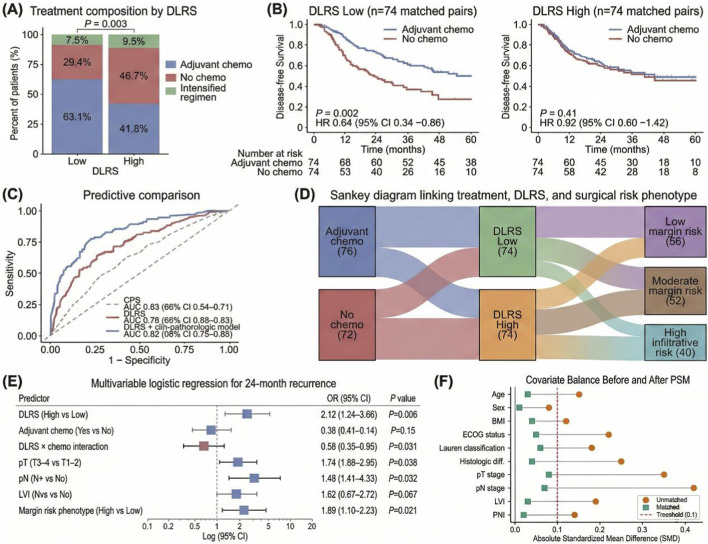
Association between the AI-driven tumor microenvironment score (DLRS) and disease-free survival (DFS) in propensity score-matched gastric cancer patients treated with or without adjuvant chemotherapy. **(A)** Stacked bar plots presenting the distribution of adjuvant chemotherapy exposure across DLRS-low and DLRS-high strata in the matched cohort. **(B)** Kaplan-Meier curves for DFS comparing patients who received adjuvant chemotherapy (n = 74) with those who did not (n = 74) within each DLRS-defined subgroup. **(C)** Receiver operating characteristic (ROC) curves comparing DLRS, conventional biomarkers, and an integrated model for prediction of chemotherapy benefit. **(D)** Sankey diagram illustrating the relationships among treatment exposure, DLRS strata, and surgery-related risk phenotypes. **(E)** Multivariable logistic regression forest plot for 24-month recurrence, showing the independent association of DLRS and its interaction with adjuvant chemotherapy. The multivariable interaction model was adjusted for predefined baseline covariates, including pathological T stage (pT3-4 vs. pT1-2), pathological N stage (N+ vs. N0), lymphovascular invasion (LVI; Yes vs. No), and margin risk phenotype (High vs. Low). **(F)** Love plot showing the absolute standardized mean differences (SMD) of baseline covariates before and after propensity score matching (PSM). Optimal covariate balance was achieved across all included clinical and pathological variables in the matched cohort (SMD <0.1 for all features across the 74 matched pairs). Figure a.

A further analysis was conducted to evaluate the potential utility of DLRS in perioperative decision-making, particularly regarding its role in identifying populations that may derive differential benefit from adjuvant chemotherapy and its association with surgery-related risk phenotypes. After propensity score matching to improve comparability between treatment groups, DLRS remained significantly associated with disease-free survival (DFS) and demonstrated evidence of treatment effect heterogeneity, supporting its use in defining treatment-related strata. Treatment exposure distributions in the matched cohort are presented in Figure 5A. Patients in the lower DLRS strata more frequently received adjuvant chemotherapy, whereas those in the higher strata had a greater proportion of non-chemotherapy or less intensive regimens. Subsequent matching procedures were applied to reduce baseline imbalance and potential selection bias, enabling comparison of treatment effects within DLRS-defined risk levels. As shown in Figure 5B, Kaplan–Meier curves illustrate DFS differences between chemotherapy and non-chemotherapy groups within each DLRS stratum. In the low-DLRS subgroup, patients receiving adjuvant chemotherapy demonstrated improved DFS relative to those without chemotherapy. In contrast, within the high-DLRS subgroup, survival curves between treatment categories showed limited separation. Predictive discrimination for chemotherapy benefit is further displayed in Figure 5C using ROC analyses, where DLRS demonstrated stronger overall performance compared with individual conventional immune or molecular biomarkers.

Associations between DLRS, treatment exposure, and surgical risk characteristics are summarized in Figure 5D. The dendrogram illustrates directional relationships among DLRS strata and categories of operative or pathological risk. Higher DLRS values were more frequently aligned with phenotypes characterized by greater infiltration or elevated margin-related risk, whereas lower scores corresponded more often to comparatively limited local aggressiveness. Multivariable adjustment results are presented in Figure 5E. After controlling for clinicopathological and therapeutic variables, DLRS remained independently associated with major adverse outcomes. Interaction signals between DLRS and adjuvant chemotherapy were also observed within the adjusted framework. Together, these findings describe the relationship of DLRS with therapeutic allocation patterns, outcome stratification, and surgery-related risk features.

### 3.4 Molecular landscape and immunotherapy sensitivity assessment

**FIGURE 6:**
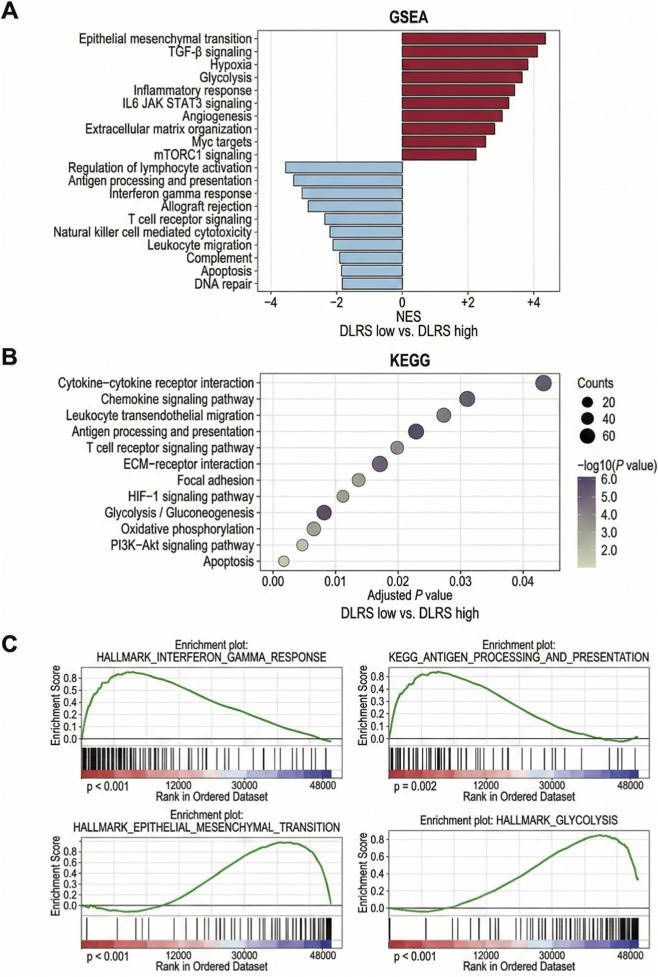
Molecular associations of the AI-driven multi-omics tumor microenvironment score (DLRS) in gastric cancer. **(A)** Bidirectional gene set enrichment analysis (GSEA) bar plot comparing normalized enrichment scores (NES) between DLRS-low and DLRS-high groups. **(B)** KEGG pathway enrichment bubble plot displaying significantly associated pathways, where bubble size represents gene counts and color corresponds to −log10(P) values. **(C)** Representative GSEA enrichment curves for selected immune-related and stromal or metabolic programs across DLRS strata.

Based on the previously established role of DLRS in tumor microenvironment classification and prognostic stratification, additional analyses examined the molecular pathway characteristics associated with DLRS and its relationship with response patterns to PD-1 immunotherapy. At the pathway level, Figure 6A presents bidirectional gene set enrichment analysis (GSEA) comparing DLRS-low and DLRS-high groups. The DLRS-low subgroup demonstrated enrichment of pathways related to immune-associated programs, including lymphocyte activation, antigen processing and presentation, and interferon-related responses. In contrast, the DLRS-high subgroup showed greater enrichment in pathways linked to stromal organization and metabolic features, including epithelial–mesenchymal transition, TGF-β signaling, hypoxia, and glycolysis. These results illustrate distinct functional program distributions across DLRS strata.

The KEGG enrichment bubble plot in Figure 6B further displays pathway distributions associated with DLRS-defined groups. Immune-related pathways, such as chemokine signaling, leukocyte transendothelial migration, and antigen processing and presentation, were more prominently represented in the DLRS-low category. Conversely, extracellular matrix interaction, focal adhesion, and metabolic pathways were more frequently observed in the DLRS-high group. The magnitude and statistical gradients shown in the Figure aindicate coordinated variation across multiple pathways rather than reliance on a single functional axis.

Figure 6C provides enrichment curves for representative gene sets to visualize the distribution of ranked signals underlying DLRS stratification. Immune-associated gene sets displayed stronger positive enrichment in the DLRS-low group, whereas matrix- and metabolism-related gene sets showed greater prominence in the DLRS-high subgroup. These plots present the relative ranking positions and enrichment intensities of selected pathways across DLRS categories.

**FIGURE 7:**
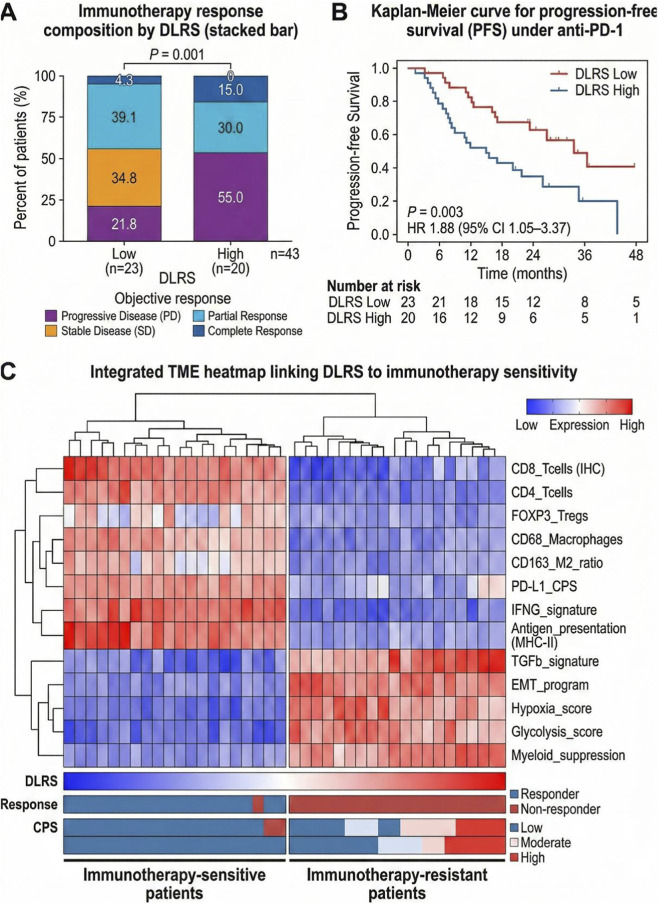
Relationship between the AI-driven tumor microenvironment score and clinical response and outcomes in a subset of gastric cancer patients treated with anti-PD-1 immunotherapy (n = 43). **(A)** Stacked bar plot showing the distribution of best overall response (Complete Response [CR], Partial Response [PR], Stable Disease [SD], and Progressive Disease [PD]) stratified by DLRS-low (n = 23) *versus* DLRS-high (n = 20). Objective response rate (ORR) and disease progression were evaluated according to the Response Evaluation Criteria in Solid Tumors (RECIST) version 1.1. **(B)** Kaplan–Meier curve for progression-free survival (PFS) comparing DLRS-low and DLRS-high groups. PFS was defined as the time from the initiation of immunotherapy to the date of first documented radiographic progression or death from any cause. Patients who were event-free at the last follow-up or lost to follow-up were right-censored. Statistical significance was determined using the log-rank test, with the Hazard Ratio (HR) and 95% Confidence Interval (CI) derived from a univariate Cox proportional hazards model. **(C)** Clustered heatmap with top dendrogram and bottom annotation tracks linking DLRS components to immunotherapy sensitivity (blue–white–red scale represents z-score normalized relative expression).

For evaluation of immunotherapy sensitivity, Figure 7A presents the distribution of clinical responses to anti–PD-1 treatment across DLRS strata. The DLRS-low group contained a higher proportion of objective responses, whereas the DLRS-high group showed increased frequencies of progressive disease or non-response. Figure 7B illustrates Kaplan–Meier curves for progression-free survival following immunotherapy. Stratification by DLRS was associated with distinct survival patterns, with earlier progression observed in the high-DLRS subgroup. These results demonstrate differences in both response status and outcome duration across DLRS-defined categories.

Figure 7C visualizes patient-level molecular patterns using an unsupervised clustering heatmap integrating DLRS with immune activity, myeloid-associated features, and stromal or metabolic programs. Clusters characterized by greater representation of T-cell–related and antigen-presentation signals were more frequently aligned with lower DLRS values. In contrast, clusters showing higher representation of myeloid and stromal or metabolic signatures were more often associated with elevated DLRS. The heatmap depicts coordinated variation among these molecular dimensions across individual samples.

### 3.5 Comparative analysis with traditional biomarkers (CPS/MSI)

Further comparisons were conducted between the AI-driven tumor microenvironment score (DLRS/TMEscore) and routinely used immunotherapy biomarkers, including PD-L1 CPS and MSI, to evaluate their relative performance in distinguishing response patterns and their potential translational utility in gastric cancer. Overall, DLRS/TMEscore demonstrated stronger separation across response categories, while additional heterogeneity remained observable within subgroups defined by conventional biomarkers.

**FIGURE 8:**
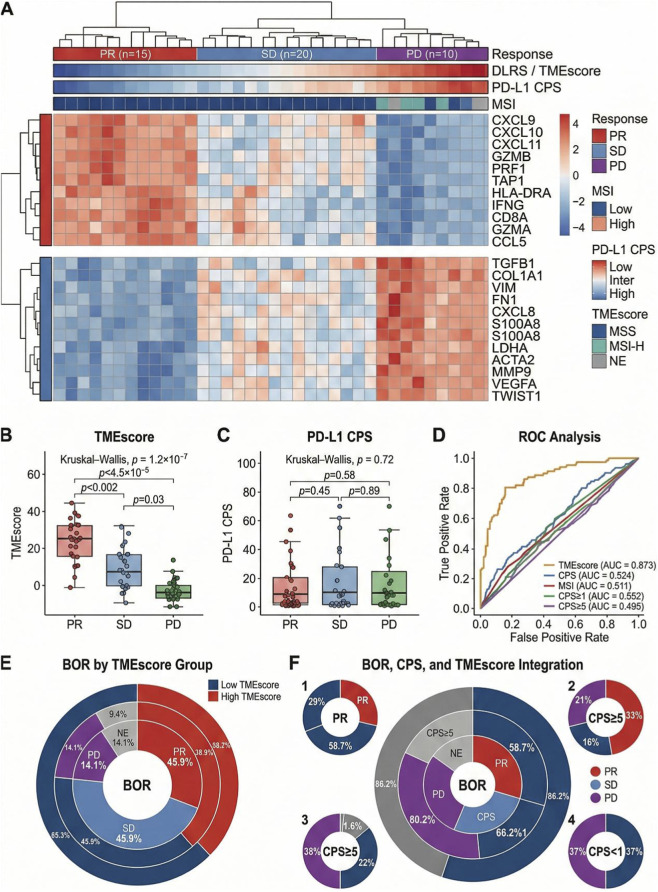
Comparative performance of the AI-driven multi-omics tumor microenvironment score (TMEscore) *versus* conventional biomarkers (CPS and MSI) in predicting immunotherapy response. **(A)** Clustered heatmap with hierarchical dendrogram, annotated by response category (PR, SD, PD) and corresponding MSI status, PD-L1 CPS, and TMEscore for each patient. **(B)** Distribution of TMEscore across PR, SD, and PD groups shown as boxplots with individual data points. Kruskal–Wallis test was used for multi-group comparison. **(C)** Distribution of PD-L1 CPS across PR, SD, and PD groups displayed in the same format. **(D)** Receiver operating characteristic (ROC) curves comparing the discriminative performance of TMEscore, CPS at specific clinical cutoffs (CPS ≥ 1 and CPS ≥ 5), and MSI status. Differences between AUCs were statistically evaluated using DeLong’s test. Detailed 95% Confidence Intervals (CIs) for each AUC are provided in Table 5. **(E)** Nested donut chart illustrating best overall response (BOR), with the outer ring stratified by TMEscore (low vs. high). **(F)** Multi-layer nested donut integrating BOR, CPS categories (CPS < 1, CPS 1-4, CPS ≥ 5), and TMEscore stratification.

In terms of molecular interpretability, the clustered heatmap in Figure 8A organizes patients according to response status (PR, SD, PD) with accompanying annotations for MSI, CPS, and TMEscore. Regions characterized by higher TMEscore values are more frequently aligned with PR classifications and are accompanied by immune-related expression patterns, whereas PD regions more often coincide with stromal or suppressive programs. In contrast, CPS and MSI annotations appear more dispersed across response clusters, indicating less concordance with the overall phenotypic grouping displayed in the heatmap.

Figure 8B presents the distribution of TMEscore across PR, SD, and PD groups using boxplots with overlaid scatter points. Differences among categories are visible, with higher values more frequently observed in PR and lower values in PD. By comparison, PD-L1 CPS distributions across response groups, shown in Figure 8C, demonstrate greater overlap, and the separation between categories is less apparent. Together, these panels illustrate differing degrees of stratification resolution between the metrics.

Regarding discriminative performance, Figure 8D displays ROC curves for TMEscore, CPS, and MSI. Across the evaluated settings, the TMEscore curve remains positioned above those of CPS and MSI, indicating comparatively stronger classification capability. CPS evaluated at different thresholds and MSI show curves closer to the diagonal reference, reflecting more limited separation performance relative to DLRS/TMEscore.

From the perspective of clinical stratification readability, the nested-ring diagram in Figure 8E depicts variations in the proportions of PR, SD, and PD between TMEscore-high and TMEscore-low groups according to best overall response criteria. Differences in composition are observable between strata. In addition, Figure 8F demonstrates that within CPS categories, further separation can still be visualized when cases are organized by TMEscore, indicating that the metric provides additional layering of response information beyond CPS or MSI alone.

Table 5 summarizes the quantitative comparisons using unified evaluation metrics, demonstrating that the AI-driven TMEscore/DLRS shows stronger discriminative performance for predicting response to immune checkpoint inhibitor therapy than alternative indicators. After threshold specification, the score also achieved improved sensitivity–specificity profiles. Beyond advantages observed in statistical metrics such as AUC, TMEscore integrates multi-omics information and microenvironment-related features into a composite index, enabling a more comprehensive representation of factors associated with immunotherapy sensitivity.

**TABLE 5:** Comparison of AI-Driven tumor microenvironment score and traditional biomarkers in predicting immunotherapy response.

Predictor	AUC (95% CI)	Sensitivity (95% CI)	Specificity (95% CI)	Youden index	Optimal cutoff	P value
AI-driven tumor microenvironment score (DLRS/TMEscore)	0.873 (0.810–0.920)	88.4% (82.1–93.2)	78.2% (71.3–84.1)	0.664	≥0.4	<0.001
PD-L1 CPS ≥ 1	0.524 (0.462–0.586)	56.2% (49.4–63.0)	60.1% (53.2–67.0)	0.164	≥1	0.32
PD-L1 CPS ≥ 5	0.552 (0.501–0.604)	62.7% (55.9–69.2)	62.4% (55.6–68.8)	0.252	≥5	0.01
MSI-H	0.511 (0.451–0.571)	53.7% (47.0–60.5)	58.5% (51.2–65.3)	0.124	MSI-H	0.29
CPS ≥ 5 and DLRS ≥ 0.4	0.873 (0.819–0.918)	87.3% (81.0–92.2)	78.7% (72.0–84.7)	0.662	CPS ≥ 5 + DLRS ≥ 0.4	<0.001

### 3.6 Clinical utility in metastatic GC and surgical outcome correlation

Based on prior validation of DLRS/TMEscore in predicting immunotherapy response and molecular subtype classification, this section further evaluated its clinical applicability in metastatic gastric cancer and explored its associations with radical surgical procedures and pathological outcomes, aiming to support translational linkage from microenvironment-related vulnerability to treatment pathway selection. Overall, DLRS/TMEscore identified subgroups with differential long-term benefit from immunotherapy in metastatic disease and demonstrated gradient relationships with surgical complexity, resection margin status, lymph node metastasis, and postoperative recovery indicators.

**FIGURE 9:**
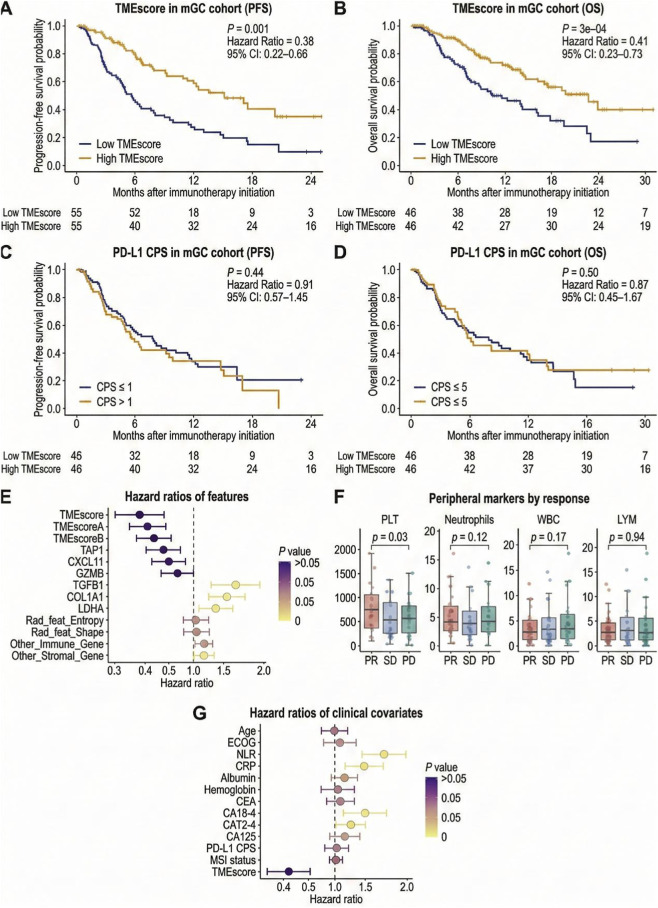
Association between the AI-driven multi-omics tumor microenvironment score (TMEscore) and survival outcomes in metastatic gastric cancer patients receiving immunotherapy. **(A)** Kaplan–Meier curves of progression-free survival (PFS) stratified by low *versus* high TMEscore, with numbers at risk displayed. **(B)** Kaplan–Meier curves of overall survival (OS) stratified by low *versus* high TMEscore, with numbers at risk displayed. **(C)** Kaplan–Meier curves of PFS according to PD-L1 CPS (≤1 vs. >1). **(D)** Kaplan–Meier curves of PFS according to PD-L1 CPS (≤5 vs. > 5). **(E)** Forest plot of hazard ratios (HRs) with 95% confidence intervals for TMEscore components; the vertical dashed line indicates HR = 1. **(F)** Boxplots with jittered individual data points showing distributions of peripheral blood biomarkers across response categories (PR, SD, PD). **(G)** Multivariable clinical covariate forest plot presenting HRs with 95% confidence intervals; the vertical dashed line indicates HR = 1.

Within the metastatic gastric cancer immunotherapy cohort, Figure 9A presents progression-free survival (PFS) and Figure 9B overall survival (OS) stratified by TMEscore. Patients in the high TMEscore group exhibited more favorable survival profiles in both PFS and OS, with stable separation of the curves and lower hazard ratios. In contrast, when stratified using conventional biomarkers (Figures 9C,D), survival differences across PD-L1 CPS categories were less distinct, suggesting limited discriminatory capacity under these thresholds. These comparisons indicate stable performance of TMEscore across complex real-world treatment contexts.

At the level of multivariable structure and interpretability, the hierarchical forest plot in Figure 9E shows that TMEscore and its principal components remained significantly associated with survival risk after adjustment. Features representing immune activation aligned with lower risk estimates, whereas variables reflecting stromal barriers, immunosuppression, or metabolic adaptation were associated with higher risk. In Figure 9F, box-and-jitter plots of peripheral blood biomarkers across response categories (PR, SD, PD) demonstrate coordinated variation with TMEscore-defined phenotypes. Furthermore, in the clinical covariate model shown in Figure 9G, TMEscore maintained an independent association with overall survival after incorporating general clinical variables, including age, inflammatory markers, and tumor biomarkers, supporting its use as a stratification factor in clinical assessment.

Beyond the immunotherapy cohort, the study further evaluated the clinical relevance of microenvironment classification in the surgical setting. Table 6 demonstrates graded differences in operative complexity and pathological risk burden across multi-omics–defined TME phenotypes. Compared with the reference phenotype, the immune-inflammation subtype was associated with a 20% longer operative time and a moderate incidence of bleeding requiring transfusion. In terms of radicality, this subtype corresponded to higher rates of R0 resection and adequate margin distance. Tumor burden indicators showed relatively fewer cases with more than six positive lymph nodes, a lower proportion of advanced pN stage, and fewer instances of vascular or neural invasion. In contrast, the stromal rejection or myeloid suppression–metabolic adaptation phenotype was associated with higher nodal involvement, more aggressive pathological characteristics, increased frequency of severe complications, and prolonged hospitalization, indicating greater perioperative burden within this group.

**TABLE 6:** Multi-omics tumor microenvironment profile correlates with surgical outcomes in gastric cancer.

Surgical outcome/Pathology metric	Immune-inflamed TME (n = 56)	Stromal-excluded TME (n = 49)	Myeloid/Metabolic-suppressed TME (n = 45)	P value
Curative-intent gastrectomy performed, n (%)	54 (96.4)	47 (95.9)	43 (95.6)	0.97
Laparoscopic/robotic approach, n (%)	21 (37.5)	14 (28.6)	12 (26.7)	0.39
Operative time, min, median (IQR)	206 (184–238)	229 (201–261)	241 (214–279)	0.004
Estimated blood loss, mL, median (IQR)	145 (95–210)	210 (140–310)	245 (165–360)	<0.001
Transfusion required, n (%)	6 (10.7)	9 (18.4)	12 (26.7)	0.048
R0 resection, n (%)	52 (92.9)	41 (83.7)	34 (75.6)	0.018
Proximal margin, mm, median (IQR)	32 (24–41)	28 (19–37)	25 (16–34)	0.006
Distal margin, mm, median (IQR)	46 (35–58)	41 (30–54)	38 (27–50)	0.041
Retrieved lymph nodes, n, median (IQR)	33 (27–41)	31 (24–39)	29 (22–36)	0.11
Positive lymph nodes, n, median (IQR)	3 (1–7)	5 (2–10)	7 (3–13)	0.003
Pathologic N stage (pN2–3), n (%)	18 (32.1)	22 (44.9)	26 (57.8)	0.013
Serosal invasion (pT4), n (%)	14 (25.0)	16 (32.7)	20 (44.4)	0.11
Lymphovascular invasion, n (%)	17 (30.4)	22 (44.9)	25 (55.6)	0.015
Perineural invasion, n (%)	15 (26.8)	19 (38.8)	23 (51.1)	0.019
Major complications (Clavien–Dindo ≥ III), n (%)	5 (8.9)	7 (14.3)	11 (24.4)	0.04
Anastomotic leak, n (%)	2 (3.6)	2 (4.1)	5 (11.1)	0.16
Postoperative length of stay, days, median (IQR)	9 (8–12)	11 (9–14)	13 (10–17)	<0.001
30-day readmission, n (%)	4 (7.1)	5 (10.2)	7 (15.6)	0.31
30-day mortality, n (%)	0 (0.0)	1 (2.0)	1 (2.2)	0.45

## 4 Discussion

This study centers on microenvironment-driven therapeutic vulnerability and develops and validates a predictive system derived from preoperative CT imaging, integrating multi-omics information to generate DLRS/TMEscore (Sun et al., 2025; Shen et al., 2025). The findings support the view that treatment-relevant differences in gastric cancer are not determined by isolated molecular events alone, but are shaped by coordinated tumor microenvironment programs, including immune activation, stromal barrier formation, myeloid-associated immunosuppression, and metabolic stress. Within this framework, DLRS/TMEscore captures these multidimensional characteristics across cohorts and provides a basis for risk stratification and the design of corresponding treatment pathways (Sang et al., 2025).

Compared with conventional biomarkers, DLRS/TMEscore shows greater stability in predicting immunotherapy response and can further refine benefit probability within strata defined by traditional indicators (Bintintan et al., 2024; Ren et al., 2025). These results suggest that reliance on PD-L1 CPS or MSI alone may be insufficient to represent the complex heterogeneity of the gastric cancer immune microenvironment, particularly in mixed or transitional states where a single marker may reflect only a partial snapshot rather than an integrated pattern. By combining immune effector signals, antigen presentation, myeloid suppression, hypoxia–metabolic features, and stromal exclusion into a composite assessment of immunotherapy sensitivity, DLRS/TMEscore provides enhanced clinical interpretability and stratification utility (Cheng et al., 2025).

The imaging manifestations are consistent with this rationale. Evidence from prior studies has shown that preoperative CT contains informative features that may assist perioperative evaluation, including assessment of residual disease burden and related operative considerations, thereby offering additional reference in clinical decision-making. Accordingly, imaging-based assessment provides a noninvasive approach for phenotype-level characterization of microenvironmental states. In the context of surgical planning, integrating such information into preoperative workflows extends decision-making beyond conventional stage-based and anatomical boundary assessment toward incorporating microenvironmental vulnerability and potential treatment resistance, supporting earlier perioperative combination-strategy design (Qi et al., 2025; Sundaram et al., 2024).

In addition, the present results indicate that DLRS/TMEscore is not limited to immunotherapy-associated benefit patterns, but is also related to variability in adjuvant chemotherapy benefit and to gradients in preoperative surgical risk and prognosis. The observed heterogeneity of adjuvant chemotherapy benefit across microenvironmental subtypes is consistent with the interpretation that treatment sensitivity is associated with baseline microenvironmental state. From a surgical perspective, subtypes characterized by stronger stromal exclusion or myeloid-associated suppression and metabolic stress tend to align with greater invasive burden and more complex perioperative risk profiles, consistent with their broader background of immune exclusion and stromal remodeling. These patterns provide risk-relevant information that may inform surgical approach selection, margin strategy, and postoperative management in a manner aligned with microenvironmental mechanisms (Hai et al., 2024; Pan et al., 2025).

From a clinical application standpoint, the results support two operational directions. First, in metastatic gastric cancer treated with immunotherapy, DLRS/TMEscore may assist in identifying patients more likely to experience sustained benefit, thereby informing prioritization and expectation management, and providing a stratified basis for research on combination strategies in lower-benefit populations (Liu et al., 2025; Deng et al., 2025). Second, for patients who are resectable or planned for radical surgery, DLRS/TMEscore may serve as a microenvironment-related indicator to support preoperative decision-making alongside imaging and anatomical assessment, contributing to individualized planning of surgical approach, resection margin strategy, and the intensity of perioperative systemic therapy.

Several limitations of this study should be acknowledged. First, the retrospective observational nature of the cohort precludes causal inferences regarding the impact of DLRS/TMEscore on surgical outcomes. While the score is highly associated with surgical complexity and margin status, extending these statistical associations to direct surgical planning (e.g., altering resection extent based on the score) requires caution. Second, although external validation was performed, the sample sizes, particularly for the immunotherapy subset, remain relatively small. Future large-scale, prospective interventional trials are mandatory to ascertain whether incorporating this multi-omics AI score into preoperative decision-making can definitively improve perioperative morbidity and long-term survival.

## 5 Conclusion

The present study investigated the tumor microenvironment (TME) in gastric cancer and developed and validated an AI-based scoring framework (DLRS/TMEscore) that integrates preoperative CT imaging with multi-omics data to characterize microenvironment-shaped therapeutic vulnerability. As demonstrated in the Results, the scoring system remained stable for prognostic stratification across cohorts, identified patient subsets with differential benefit from adjuvant chemotherapy and anti–PD-1 therapy, and provided incremental discriminatory value beyond conventional biomarkers such as CPS and MSI. In addition, DLRS/TMEscore was associated with indicators of surgical complexity, resection margin status, lymph node involvement, and postoperative recovery. These findings support the potential role of this index in informing preoperative risk stratification and provide an interpretable basis for perioperative risk assessment and individualized comprehensive treatment strategies in gastric cancer (Ji et al., 2024; Li et al., 2025).

## Data Availability

The raw data supporting the conclusions of this article will be made available by the authors, without undue reservation.
